# Improving research integrity: a framework for responsible science communication

**DOI:** 10.1186/s13104-022-06065-5

**Published:** 2022-05-15

**Authors:** Ilinca I. Ciubotariu, Gundula Bosch

**Affiliations:** 1 Department of Biological Sciences, Purdue University, West Lafayette, IN 47907 USA; 2 Department of Molecular Microbiology and Immunology, Johns Hopkins Bloomberg School of Public Health, R3 Center for Innovation in Science Education, Baltimore, MD 21205 USA

**Keywords:** Research integrity, Rigor, Responsibility, Scientific communications training, Institutional graduate education programs

## Abstract

Research integrity, an essential precept of scientific inquiry and discovery, comprises norms such as Rigor, Reproducibility, and Responsibility (the 3R’s). Over the past decades, numerous issues have arisen that challenge the reliability of scientific studies, including irreproducibility crises, lack of good scientific principles, and erroneous communications, which have impacted the public’s trust in science and its findings. Here, we highlight one important component of research integrity that is often overlooked in the discussion of proposals for improving research quality and promoting robust research; one that spans from the lab bench to the dissemination of scientific work: responsible science communication. We briefly outline the role of education and institutions of higher education in teaching the tenets of good scientific practice and within that, the importance of adequate communications training. In that context, we present our framework of responsible science communication that we live by and teach to our students in courses and workshops that are part of the Johns Hopkins Bloomberg School of Public Health R^3^ Center for Innovation in Science Education.

## Introduction

Science has a credibility problem. The underlying issues are multi-factorial such as inadequate training in rigorous research methods, irreproducibility of results, logical fallacies and statistical mistakes during data analysis and interpretation, erroneous communication, sloppy literature outputs, and outright misconduct (e.g., [1–6]). The result is an ongoing pandemic of retractions [7–10]. That in turn can undermine public confidence in research outcomes and transparent policy [11–13], along with societal factors such as geography or culture [14]. It is of utmost importance to properly train the next generation of scientists and protect the integrity of the central principles of scientific inquiry and discovery.

This commentary discusses the role of institutional graduate programs in promoting good research practice through teaching the core values of reliable science, while at the same time, focusing on a framework for embracing responsible scientific communication.

## Main text

### The role of institutional graduate education programs in promoting research integrity

There have been numerous calls to reform biomedical and health science education at the graduate level [15–17]. Yet frequently, established programs fail at conveying skill training in fundamental key competencies that are crucial to preparing graduates for the complexities of present-day workplaces: critical, interdisciplinary, and creative thinking [18]. At the R^3^ Center for Innovation in Science Education (R^3^ISE) at the Johns Hopkins Bloomberg School of Public Health (BSPH), we develop interdisciplinary graduate- and post-graduate level programs and resources that emphasize precisely these competencies by taking a unique approach to scientific competency training. In the United States and through a growing international network of partner institutions, we spearhead reform efforts to educate future practitioners across the science and health disciplines in applying the philosophical foundations of science to their research; engaging in interdisciplinary collaboration; communicating effectively; and committing to the highest standards of scientific integrity. Program participants are trained in the epistemology of reliable evidence generation; applied logic; practical ethics; robust methodological approaches; and effective communication, all based on the three “R” norms of good science – Responsibility, Rigor, and Reproducibility (R^3^) [18].

### Enhancing research integrity through responsible communication

In present times, rapidly evolving scientific evidence, mixed messaging, and sometimes misinformation, can influence the public’s fluctuating trust in science and its products [19–21]. Trust needs to be rebuilt where it was lost [22, 23], and maintained where it began to grow [24] to help increase acceptance of and adherence to public health guidelines [25, 26]. Strictly rigorous and reproducible research practices are herewith a *sine qua non*, but the appropriate and truthful *communication* of science, its methods, results and pitfalls, is just as important for enhancing research credibility. It is a scientist’s duty to devote appropriate efforts toward good science communication. Known mistakes in this field—common among amateur and professional science communicators alike—are incomplete background research, hasty assumptions, factual misrepresentations, or overstatements in social media outlets, newspapers, or interviews. In a societal climate where a considerable portion of news consumers in all parts of society is inclined to listen more to rumors and unsubstantiated claims rather than rigorous scientific evidence, a lack of responsible science communication opens the floodgates for the plagues of mis- and dis-information even wider [27, 28]. The current times are thus a continuous existential reminder of our duty to provide the public with clear and actionable information.

Appropriate science communication can and needs to adhere to good practice standards. While we concur that scientific communication is not evaluated with the same metrics as the appropriate conduct of science itself [29], we agree with those members of the science community who call for high quality standards in scientific communication [30–33]. Many current coaching efforts to help scientists become better communicators focus on doubtlessly important stylistic and strategic questions, e.g., goal setting, audience orientation, argumentation structure, choice of language, and persuasive messaging [34]. More comprehensive perspectives are needed in tandem with the growing appreciation for the roles of training in developing competences for science communication [35–37]. Furthermore, we claim that there is more to it, namely a fundamental, ethical habit of mind: *Responsibility*.

### The notion of responsibility in the context of science communication

Communicating science *responsibly* implies that scientists must deliver more than jargon. If we view non-scientists as empty buckets to be impressed by and filled with sophistical information, akin to the well-known deficit model of communication with a one-way flow of information from experts to laypersons [38], we will rightfully be perceived as arrogant and create alienation rather than alliances. There has been much debate around the deficit model [39], which initiated a push to incorporate dialogue, context, and public engagement in scientific communication [40, 41]. Members of the public are essential in scientists’ efforts to disseminate truthful information. It is important for scientists to actively reach out to the public, instead of merely talking to other specialists [42]. The words of the late Stephen Hawking come to mind, who stated that “Not only is it important to ask questions and find the answers; as a scientist I felt obligated to communicate with the world while we were learning.” Hawking’s wisdom reminds us that researchers need more confidence to explain that science is not a simple, clear-cut issue. Scientific facts are not easy to convey. They are subject to an ever-evolving process that includes constant learning, critical evaluation of new evidence, and revision of existing views and theories. The pitfalls of science such as reproducibility problems, sloppy literature, at times dubious review processes, and a rising number of retracted articles can pose a true challenge to bringing the actual nature of science across: namely the quest for truth, while maintaining the highest standards of integrity. The consequences are—not rarely—citizens who distrust the scientific process and its practitioners.

These expectations may seem understandably daunting. Most scientists have never received a formal education in this domain. Without a guiding framework that helps master challenging situations, many scientists may avoid commenting on the ambiguities that are inherent to the scientific process [43]. They might react helplessly when consulted to contradict misinformation or become defensive when asked to comment on cases of sloppy science or even misconduct. Such insecurities, however, give way to conspiracy theorists and spreaders of intentional falsehoods. They can spawn denial, at times even hostility, among many members of society who feel uneasy with the reality that evidence generation in science is not perfect. As scientists, we must learn to confidently explain -and not apologetically defend- that science is a dynamic process involving trial and error that does not allow quick yes-or-no answers [44].

There is no time to lose. Too long have we scientists been sitting comfortably in our academic ivory towers, hoping that some talented science writer will do the communications job with the “world out there” for us. It really is upon us to improve and prevent the spread of misinformation and misconceptions, an issue that is extremely relevant amidst the current pandemic [45].

**Fig. 1:**
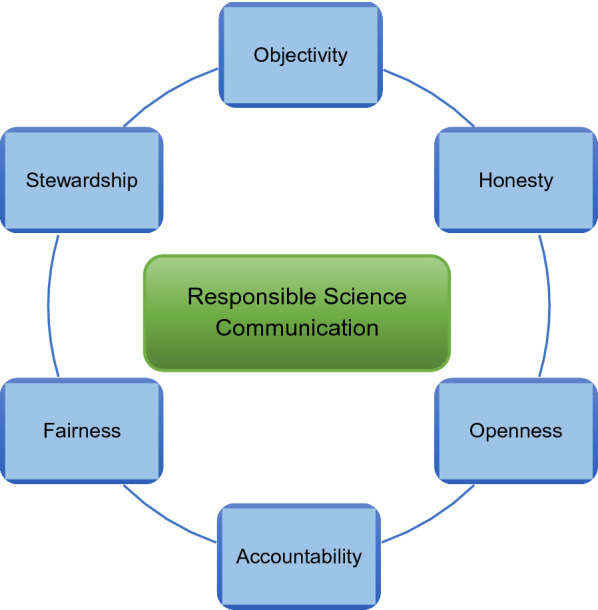
Responsible Science Communication Framework. This framework applies value-based recommendations on ethical research conduct to practical science communication

In what follows, we outline some general, value-based guidelines (Fig. 1) built on established ethical principles [46–48] widely accepted in the scientific community that helped us, our students, and colleagues at the BSPH R^3^ Graduate Science Program [18, 49] in our science communications training and practice efforts. We appreciate the parallels between this and the important Responsible Research and Innovation policy framework set forth by the European Commission, to tackle societal challenges through an engagement of public and responsible actors in science and innovation [50, 51]. Similarly, our program puts a strong focus on the ethical underpinnings of scientific conduct of which responsible communication is an integral part. We are not claiming that our approach is the *ne plus ultra*. Rather, it is meant as a starting point to build upon, since communication is a lifelong learning process.

#### Objectivity

In science and science communication alike, “certain kinds of motivation, position, material interests, field of specialty, prominence, or other factors should not influence a researcher’s actions” and decision making [46]. This includes conflicts of interest, implicit and explicit biases, and unintentional yet still questionable research and communication practices [48, 52] to which every human being can fall victim. A responsible science communicator should be aware of those risks. Recognizing the need for constant self-improvement, scientists should do their best to develop a habit of critical self-reflection, good listening, and actively seeking feedback from peers and the public.

#### Honesty

Needless to say, intellectual honesty is at the center of doing good science—and so is honest science communication. Science practitioners have a role model function in society and must live up to it. Honesty implies truthfulness and epistemic humility, i.e., staying true to the facts that are known; realizing the limits of one’s expertise by avoiding overstatements; and recognizing gaps and ambiguities in the knowledge base. For instance, honest communication and not withholding conflicting information about vaccines can increase trust in science [53]. Acknowledgement of findings that do not fit with one’s original hypothesis can mean good things, i.e., steps to a new understanding, and can be communicated accordingly. Following wise advice attributed to Confucius, committing a mistake without correction is like committing another mistake.

#### Openness

Responsible science communication describes facts and realities, not what we desire to see or what sounds opportune. In an era of Open Science [54], scientists ought to be as transparent as possible with regard to providing open access to all current data, the methods used to obtain the data, as well as valid conclusions given the evidence available at the time [55]. This pandemic clearly demonstrates the urgent need for increasing scientific cooperation through universal access to scientific progress, which has the power to unite nations [56]. Practicing openness in science communication also includes revealing potentially confusing data or mistakes, as trial and error is an integral part of the scientific process.

#### Accountability

Closely related to the value of openness is the notion of accountability. It implies that researchers have an obligation to explain their work and justify their methods, results, and interpretations [46]. Rigorous conduct of science is of course essential, albeit not enough for accountability to the public. There are a variety of ways by which science professionals can hold themselves accountable to broad audiences. Many journals, grant agencies or conference organizers already request abstracts in lay-terms. Upon publishing preprints, authors could provide non-technical narratives of their findings through virtual open houses, websites, podcasts, community-science forums, OpEds, social media updates or press releases. While unfamiliar at first, those communications formats can provide invaluable opportunities to interact with the sovereign that should not be missed and henceforth enhances research integrity.

#### Fairness

The notion of fairness includes “[…] impartial treatment [and the] lack of favoritism toward one side or another” [57]. To live up to this standard, we need to put value on clear, accessible language that does not discriminate and allows equal opportunities for participation; chooses dialogue over dominance; shows respect and mindfulness in our choice of words; demonstrates appreciative audience orientation and receptiveness to questions; and accepts critique and welcomes others’ viewpoints made in good faith.

#### Stewardship

Good stewardship in the context of science communication implies that we humbly understand our capacities as scientists as a privilege that is made possible for us by members of the public in the expectation that we make the best use of resources we are given. We are paid for thinking and pursuing interesting questions. Those who fund us, namely the taxpayers, should receive something back outside of research results. Scientists are serving the common good and thus should view intelligible communication as an integral part of their job, their training efforts, as well as their own, continuing education.

## Outlook

There is great power in the ethical core values of good research practice, and we advocate for using them as the basis for our communication efforts as well. Certainly, persuasiveness in expression, careful choice of wording, in combination with effective messaging are integral parts of good communications crafts (wo)manship. Yet, eloquence and elegance in one’s rhetoric cannot replace a critically-thinking, ethics-oriented mindset. Responsibility toward the trust that the public puts in us should be the compass in a scientist's fight against miscommunication, misinterpretation, misstatements, falsehoods, and pseudoscience. We owe it to society. Echoing the words of Atul Gawande [58], when “you become part of the scientific community, arguably the most powerful collective enterprise in human history, […] you also inherit a role in explaining [the nature of science] and helping it reclaim territory of trust at a time when that territory has been shrinking.”

## Data Availability

Not applicable.

## Abbreviations

BSPH: The Johns Hopkins Bloomberg School of Public Health
R^3^: Rigor, Responsibility, and Reproducibility
R^3^ISE: R^3^ Center for Innovation in Science Education
